# Research Progress of the Application of Hypothermia in the Eye

**DOI:** 10.1155/2020/3897168

**Published:** 2020-12-15

**Authors:** Lei Xi

**Affiliations:** Division of Experimental Vitreoretinal Surgery, Centre for Ophthalmology, University of Tübingen, Schleichstrasse 12/1, 72076 Tübingen, Germany

## Abstract

Hypothermia is widely used in the medical field to protect organs or tissues from damage. Different research fields have different explanations of the protection mechanism of hypothermia. Hypothermia is also widely used in the field of ophthalmology, for example, in the eye bank, the preservation of corneal tissue and the preservation of the eyeball. Low temperature can also be applied to some ophthalmic diseases, such as allergic conjunctivitis, retinal ischemia, and retinal hypoxia. It is used to relieve eye symptoms or reduce tissue damage. Hypothermic techniques have important applications in ophthalmic surgery, such as corneal refractive surgery, vitrectomy surgery, and ciliary body cryotherapy for end-stage glaucoma. Hypothermia can reduce the inflammation of the cornea and protect the retinal tissue. The eyeball is a complex organ, including collagen tissue of the eyeball wall and retinal nerve tissue and retinal blood vessels. The mechanism of low temperature protecting eye tissue is complicated. It is important to understand the mechanism of hypothermia and its applications in ophthalmology. This review introduces the mechanism of hypothermia and its application in the eye banks, eye diseases (allergic conjunctivitis, retinal ischemia, and hypoxia), and eye surgeries (corneal transplant surgery, corneal refractive surgery, and vitrectomy).

## 1. Introduction

The application of hypothermia in medicine has a long history [1]. Hypothermia therapy has a wide range of applications in many fields of medicine, such as severe stroke, brain trauma, and neonatal hypoxic-ischemic encephalopathy. Hypothermia therapy has been a potentially important clinical treatment method. In addition, hypothermia therapy can preserve living tissue and prolong the survival time of organs to a certain extent, but the mechanism is still unclear.

In the field of ophthalmology, hypothermia is mainly used for preservation of corneal and scleral tissues in the eye bank, local cooling during eye surgery to protect the corneal endothelium and retinal tissue, cryotherapy in intracapsular cataract extraction surgery, and cyclocryotherapy in the treatment of glaucoma. However, there is still no uniform temperature standard for the method of preserving the cornea in the eye bank [2]. On the other hand, hypothermia may decrease intraocular bleeding volume and reduce fibrin production and postoperative inflammation [3]. Hypothermia can reduce the metabolism and the levels of VEGF secreted by cultured retinal pigment epithelium (RPE) [4]. So, targeting the RPE metabolism may produce a strategy for the research in choroidal neovascularization (CNV) or age-related macular degeneration (AMD). The eyeball organ contains not only the cornea and sclera which are rich in collagen tissue but also the nerve-rich retina and optic nerve. The basic research of hypothermia in ophthalmology focused on the impact of hypothermia on the corneal endothelium and the impact of hypothermia on the corneal transplantation (including corneal endothelial transplantation) and the survival of corneal endothelial cells after corneal transplantation [5–7]. Also, the survival characteristics of inner and outer retinal cells and the protective mechanism on the retina nerve under hypothermia are investigated [8, 9]. In this review, the protective mechanism of hypothermia will be introduced, and the applications of hypothermia in ophthalmology are summarized.

## 2. Hypothermic Protection Mechanism

### 2.1. Effect of Hypothermia on Cell Metabolism

#### 2.1.1. Reduce Oxygen Consumption

Mitochondria are the sites of oxidative metabolism and synthesize adenosine triphosphate (ATP) to provide energy for cells. The normal function of mitochondria requires the participation of a series of enzymes, but the activity of many enzymes in the cell decreases under hypothermia.

Hypothermia might preserve the ATP levels, slow the cellular process, and reduce metabolic demands. Simkhovich et al. investigated whether moderate reduction in regional myocardial temperature protects the myocardium from sustained ischemic insult by using a rabbit animal model. They thought that hypothermia preserved ATP and glycogen stores in the ischemic area. The protective mechanism of local hypothermia could be mediated through the preservation of critical energy metabolism [10]. Choi et al. explored the effect of hypothermia on the ischemia-reperfusion (IR) kidney. The results showed that hypothermia preparation protected against renal IR injury by inducing extracellular regulated protein kinase (ERK) phosphorylation and HIF1 activation in the IR kidney. Inhibition of ERK phosphorylation aggravated apoptosis and oxidative stress in hypothermic IR kidney. ERK phosphorylation may play a role in protecting against IR injury [11].

Hendriks et al. explored mitochondrial oxygen consumption and reactive oxygen species (ROS) production and scavenging capacity in porcine kidneys by mimicking the transplantation procedure. The results showed that mitochondrial oxygen consumption decreased more than ROS production at hypothermic temperatures. However, compared to the much stronger decrease in oxygen consumption and mitochondrial membrane potential during hypothermia, the H_2_O_2_ production is relatively higher. Malondialdehyde (MDA) affects the mitochondrial respiratory chain and key enzyme activities. The level of MDA (a marker of ROS-induced lipid peroxidation) was increased in cultured human epithelial kidney cells (HEK293) at a low temperature. This suggested that a low temperature might decrease the overall antioxidant capacity of the cells and then results in increased MDA. On the other hand, the protein levels of the mitochondrial scavenger manganese superoxide dismutase (MnSOD) were decreased in cooled HEK293 cells. MnSOD is an antioxidant metalloenzyme in the organism. During hypothermia, the ROS scavenging enzymes may get depleted. So, reducing the formation of H_2_O_2_ and MDA is also an important target of hypothermia [12].

One study found that hypothermia could reduce the metabolism and the levels of vascular endothelial growth factor (VEGF) secreted by the cultured retinal pigment epithelium (RPE). However, pharmacological agents which could slow down the metabolism could not reduce the VEGF secretion [4]. They suggested that reducing retinal oxygen demand may be a way to mitigate ischemia and retinovascular disease. Targeting the RPE metabolism may produce a strategy for the research in choroidal neovascularization (CNV) or age-related macular degeneration (AMD). But whether long-term hypothermia can bring about other side effects on visual function remains unknown.

#### 2.1.2. The Effect of Hypothermia on the Normal Function of Mitochondria

Hypothermia results in a decrease in the activity of the respiratory chain complexes in mitochondria. Dubinin et al. reported that hypothermia is accompanied by an increase in the content of unsaturated fatty acids in the lipid composition of liver mitochondrial membranes. This change leads to the induction of Ca^2+^-dependent mitochondrial pore (mitochondrial permeability transition pore (mPTP)) opening. mPTP can cause the impairment of ATP synthesis, swelling of the mitochondrial matrix, rupture of the outer membrane, and release of cytochrome c and other proapoptotic proteins [13]. The balance between ATP production and ATP consumption in mitochondria is important for the survival of cells under hypothermic conditions. Hendriks et al. reported that the nonhibernator-derived kidney cell line HEK293 is vulnerable to hypothermic stress conditions, resulting in a loss of mitochondrial membrane potential (MMP) and ATP production and cell death in an in vitro hypothermic model. Different species' mitochondria have different adaptability to hypothermia [14]. Another showed that mild hypothermia (32°C) caused significant increases in the mitochondrial membrane potential, the adenosine triphosphate content, and mitochondrial respiration during ischemia-reperfusion injury in ex vivo liver transplantation [15].

#### 2.1.3. The Protective Effect of Hypothermia on Mitochondria

Hypothermia affects the normal function of mitochondria and also has a certain protective effect on mitochondria. The protective mechanism of hypothermia on mitochondria is still unclear. Pamenter et al. examined how mitochondrial function is impacted by temperature in a saponin-permeabilized mouse brain at 28°C and 37°C. The results showed that hypothermia might improve mitochondrial function in the hypoxic brain by energy conservation and enhancing cellular viability. Therapeutic hypothermia might provide neuroprotective benefits to hypoxia-intolerant species by enhancing the efficiency of mitochondrial respiration [16]. Peroxisome proliferator-activated receptor is a group of nuclear receptor proteins that function as transcription factors to regulate gene expression. Peroxisome proliferator-activated receptor gamma coactivator 1-alpha (PGC-1*α*) is a multifunctional transcription regulator, widely involved in mitochondrial biosynthesis, energy metabolism, glucose and lipid metabolism, and other processes. Zhang et al. thought that hypothermia upregulated the expression of long noncoding RNA-UIHTC (upregulated in hypothermia-treated cardiomyocytes, NONHSAT094064), which protects against myocardial infarction through improving mitochondrial function via PGC-1*α* [17].

Huang et al. found that hypothermia could restore mitochondrial NADH cytochrome c reductase activity and protect mitochondrial integrity by reducing the mitochondrial permeability transition pore (mPTP) opening. Also, the reduced mPTP can prevent the uncontrolled release of cytochrome c and activation of damage cascades [18]. Signal transducer and activator of transcription 3 (STAT-3) is a transcription factor capable of mediating a cardioprotective effect by activating antiapoptotic genes and downregulating proapoptotic genes [18–20]. In this study, they also found that phosphorylated STAT-3 (Tyr705) was more highly expressed in both the cytosol and mitochondria under hypothermic conditions. Mitochondrial STAT-3 can be activated by hypothermia treatment and mediate hypothermia-related protection [18]. Khaliulin et al. investigated whether short-term hypothermic perfusion and rewarming could protect hearts against ischemic/reperfusion injury. They suggested that the protective effect could be explained by decreased reactive oxygen species (ROS) accumulation and a reduction in mitochondrial permeability transition pore (mPTP) opening and improved contractile function [21].

### 2.2. Hypothermia and Cold-Inducible RNA-Binding Protein

#### 2.2.1. The Protective Mechanism of Hypothermia and Cold-Inducible RNA-Binding Protein

Cold-inducible RNA-binding protein (Cirbp) and cold-inducible RNA-binding protein motif 3 (RBM3) are the two main cold-induced proteins studied for the neuroprotection. Hypothermia therapy has been proven neuroprotective in patients suffering from neural injuries. However, the mechanism of Cirbp in hypothermia protection is still unclear.

Rey-Funes et al. reported that hypothermic condition of 8°C could protect the retinal damage from traumatic neuropathy and prevent vision loss, as measured by electroretinography in rats. They suggested that the molecular mechanism responsible for the protection seemed to include the upregulation of two cold-shock proteins (CSPs), RBM3 and CIRBP [22]. RBM3 is a crucial factor which mediates hypothermia-induced neuroprotective effects by inhibiting the mitogen-activated protein kinase (MAPK) signaling of p38, JNK, and ERK. One study found that hypothermia (32°C) significantly reduced the apoptosis induced by rotenone (ROT) in human neuroblastoma SH-SY5Y cells [23]. Ushio and Eto reported that RBM3 expression was upregulated at the transcriptional level but not at the posttranscriptional level in cells under hypothermia. A critical mediator of mild hypothermia, the transcription factor NF-*κ*B p65, is phosphorylated at Ser276, upregulates the RBM3 gene expression, and then suppresses the induction of apoptosis during mild hypothermia. Conversely, a potent and specific inhibitor is the caffeic acid phenethyl ester (CAPE), which suppresses the translocation of NF-*κ*B p65 and resulted in decreased levels of RBM3 mRNA and protein and increased the incidence of apoptosis under hypothermic conditions. However, how RBM3 suppresses the induction of apoptosis remains to be investigated [24].

The effect of hypothermia on the metabolic rate, reducing the generation of radicals, reducing inflammatory response, and inhibiting neuron cell apoptosis, may play an important role in the neuroprotection [25].

#### 2.2.2. Effects of Cold-Shock Protein Levels

RBM3 is implicated in cell survival. Blocking RBM3 expression in neuronal cells by specific siRNA significantly diminished the neuroprotective effect of hypothermia. Together, inducing RBM3 expression endows neuronal cells with more resistance against induction of apoptosis, even under euthermic conditions (37°C). Chip et al. indicated that the degree of neuronal apoptosis is inversely related to the level of RBM3 expression [26]. Zhu et al. found that hypothermia attenuated apoptosis in wild-type (WT) mice in both subventricular zone (SVZ) and subgranular zone (SGZ) neural stem/progenitor cells (NSPCs). In RBM3 knockout (KO) mice, hypothermia only marginally attenuated apoptosis. The results suggested that RBM3 partially mediated hypothermic cytoprotection in NSPCs. RBM3 limits hypoxic ischemia- (HI-) induced apoptosis at least partially mediating the protective effects of cooling [27]. Peretti et al. injected lentiviruses LV-RBM3 in hippocampi of mice to increase the production of RBM3. The results showed that the increased RBM3 expression protected against synapse loss in prion disease, restored synaptic transmission, and prevented behavioral deficits. In contrast, knockdown of RBM3 by injecting lentiviruses LV-shRNA-RBM3 accelerated synapse loss, led to memory and behavioral deficits, accelerated neuronal loss, and shortened survival. However, whether cooling induces the loss of synaptic contacts and whether the transmission of synapses is normal after returning to normal body temperature also need to be investigated [28].

## 3. Hypothermia and Endoplasmic Reticulum

The endoplasmic reticulum (ER) is a membranous structure inside the cell. ER stress often occurs in the presence of harmful factors. The effect of hypothermia on the ER mainly focuses on the control of ER stress.

Therapeutic hypothermia (TH) (core temperature, 33-35°C) protects the endoplasmic reticulum from ultrastructural changes and reduces apoptosis of the cerebral cortex following cardiac arrest (CA) in experimental pigs. The reduction of neuronal apoptosis might occur through suppression of ER stress [29].

Hypoxia induces ER stress and activates unfold protein response, resulting in apoptosis by activating CCAAT-enhancer-binding protein homologous protein (CHOP). Global cerebral ischemia induced upregulation of CHOP and endoplasmic reticulum oxidoreductin-*α* (Ero1-*α*) in rat hippocampi, while induced hypothermia (31°C) attenuates CHOP and augments Ero1-*α* expression in the PC12 cell line [30]. The protective mechanism of hypothermia preventing apoptosis may be through a downregulation of the proapoptotic factor CHOP and an increase in Ero1-*α* expression. Another study on the effect of hypothermia on the intracerebral hemorrhage rat model showed that hypothermia induced a decrease in the expression of the ER stress-associated protein, glucose-regulated protein (GRP78), CHOP, and phosphorylate eukaryotic initiation factor 2 (p-eIF2) [31]. Hypothermia prevented the neuron apoptosis which might be through decreasing the ER response.

Mild hypothermia (33°C) could reduce prolonged ER stress-induced apoptosis of neuronal cells in a mouse-controlled cortical impact injury model. The protective effect of therapeutic hypothermia might be through attenuating protein kinase R- (PKR-) like endoplasmic reticulum kinase (PERK) and inositol-requiring enzyme 1alpha (IRE-1*α*) pathway marker protein expression [32].

ER stress response was increased in cell cultures with transient RNA-binding motif protein 3 (RBM3) knockdown and in hippocampal organotypic slice cultures from RBM3 knockout mice. The results showed that RBM3 played a key role in blocking the ER stress response. RBM3 inhibited PRKR-like ER kinase (PERK) phosphorylation through cooperation with the nuclear factor 90 (NF90), ultimately reducing apoptosis [33].

However, ER stress and oxidative stress usually accentuate each other; antioxidants combined with ER stress inhibitors may be more protective. Targeting the source of reactive oxygen species (ROS) production rather than ROS themselves may offer better neuroprotection than classical antioxidants [34].

## 4. Hypothermia and Inflammation

Hypothermia plays a role through a variety of mechanisms such as reducing metabolic demand, upregulating of two cold-shock proteins, inhibiting free radical release, and anti-inflammation.

The cellular mechanism of the neuroprotective effect of therapeutic hypothermia (TH) (33.5°C) has not yet been fully elucidated. Kimura et al. found that hypothermia (33.5°C) suppressed microglial inducible nitric oxide synthase (iNOS) and proinflammatory cytokine expression such as TNF-*α* and IL-1*β*. Also, hypothermia attenuated neuronal damage via inhibition of microglial activation [35]. Serdar et al. found that microglia were early key mediators of the inflammatory response following inflammation-sensitized hypoxic ischemic (HI) brain injury. The microglia polarize into a predominant proinflammatory phenotype after 24 hours of HI [36].

Zhao et al. evaluated the neurovascular protective potential of pharmacological hypothermia induced by the neurotensin receptor 1 agonist HPI-201 after severe ischemic stroke in adult C57BL/6 mice. Stroke mice receiving the hypothermic treatment showed a lower neurological severity score and a decreased expression of inflammatory factors including TNF-*α*, MMP-9, and IL-1*β*. Hypothermia is protective for neurovascular cells [37]. Xiao et al. reported that mild hypothermia pretreatment attenuated inflammatory response against liver ischemia and reperfusion (IR) injury via the phosphoinositide 3-kinase (PI3K)/protein kinase B (AKT)/FOXO3a pathway. Hypothermia pretreatment could protect the liver by inhibiting the degree of apoptosis and inflammation [38]. Hypothermic machine perfusion significantly inhibited inflammatory factor expression and decreased the apoptosis and necroptosis of renal cells during ischemia/reperfusion injury by upregulating the expression of A20 [39].

Drapalova et al. explored the influence of deep hypothermia on systemic and local inflammation, adipose tissue hypoxia, and adipocytokine production. The results showed that deep hypothermia connected with an anoxic phase during cardiosurgical operation reduced the development of systemic operation-induced inflammation and delayed the local adipose tissue inflammation. They also found that deep hypothermia could delay or suppress mRNA expression of inflammation- and oxidative stress-related factors in adipose tissue [40]. However, hypothermia followed by rapid rewarming augmented proinflammatory response and aggravated neuronal loss and exacerbated blood-brain barrier breakdown in a rat stroke model [41]. The inflammatory factors may be potential causes of tissue damage after rapid rewarming. Hypothermia followed by slow rewarming can inhibit the expression of proinflammatory cytokines.

The major mechanism of hypothermic neuroprotection might be due to its suppression of proinflammatory cytokine expression and anti-inflammatory property [42]. Hypothermia significantly suppressed inflammatory-related molecules including IL-1*β* and osteopontin (OPN). This means that suppression of neuroinflammation primarily contributes to neuroprotection [43]. The therapeutic time window, such as when to start hypothermia treatment and how long hypothermia treatment should be maintained, still needs further study.

## 5. Hypothermia and Microglia

Microglia is a highly plastic cell with coexisting diverse phenotypes, polarization of M1 and M2. Polarized M1 microglia are considered to be proinflammatory, whereas polarized M2 microglia are more closely linked to anti-inflammatory factors. Therapeutic hypothermia (TH) inhibited the M1 polarization of microglia and reduced the expression of inflammatory factors such as TNF-*α*, IL-1*β*, and iNOS in stroke mouse model [44]. They suggested that TH suppressed inflammatory responses via regulation of M1/M2 microglial activation after stroke, thus having the protective effect. Truettner et al. evaluated the effects of posttraumatic hypothermia on phenotype patterns of microglia. Early hypothermia reduced M1-associated gene expression while increasing M2-associated gene expression after traumatic brain injury in a rat model. Therapeutic hypothermia can alter the M1/M2 phenotype balance and have a significant effect on the reduction of an inflammatory state after traumatic lesion [45]. Future studies are required to find a suitable targeted hypothermic temperature and optimal therapeutic windows for different conditions.

Hypothermia exerted the neuroprotective effect against ischemic brain injury which might occur by regulating glial pathways, such as glutamate signaling, cell death, and stress response. Also, hypothermia modulated the specific proteins in glia and subsequent phenotypic changes which might form the basis of protective effects [46]. Another study reported that hypothermia upregulated glial fiber acidic protein (GFAP) by decreasing caspase-3 expression and increasing Bcl-2 expression to inhibit cell apoptosis and reduce brain injury [47]. So, investigating the effects of hypothermia on reactive glial cells at the molecular level can expand our understanding of the neuroprotective effects of hypothermia.

Xiong et al. revealed that hypothermia enhanced mature oligodendrocytes and promoted the regeneration and maturation of oligodendrocyte precursor cells (OPCs) as well as rescuing animal behavior associated with decreased O4^+^ late OL progenitors' (preOLs) accumulation and degeneration after hypoxic-ischemic injury [48]. Toriuchi et al. assessed the expression of erythropoietin (EPO) and hypoxia-induced factor (HIF) in cultured astrocytes derived from the rat cerebral cortex and cultured under oxygen/glucose deprivation (OGD). The results showed that hypothermia (33.5°C) upregulated both EPO and protein expression and upregulated EPO expression could suppress neuronal apoptosis [49]. They reported that EPO expression was predominantly upregulated due to increased HIF-2*α* protein expression, and hypothermia was found to stabilize HIF-EPO signaling in astrocytes.

Autophagy is a phagocytosis of its own cytoplasmic proteins or organelles, thereby achieving the cell's own metabolic needs and the renewal of certain organelles. Moderate hypothermia can reduce the number of activated microglia by inhibiting autophagy and promoting apoptosis in a traumatic brain injury (TBI) model [50]. However, how moderate hypothermia specifically affects the autophagy and apoptosis of Müller glia after ischemia is not very clear.

## 6. Hypothermia and Glutamate

Glutamate is one of the toxic mediators inducing neuronal injury under ischemic conditions. Wang et al. investigated the mechanisms mediating glutamate release during brain ischemia-reperfusion injury under hypothermic conditions. The results showed that hypothermia reduced the levels of extracellular glutamate induced by oxygen-glucose deprivation (OGD) injury via downregulation of glutamate transporter (GLT-1), which is the response for the clearance of extracellular glutamate. But the mechanisms that contribute to this process are not clear [51]. The astrocytes have been found to control the release of glutamine. However, astrocytes can also reverse the transport of glutamate into the extracellular space during the early stages of ischemia.

Nomura et al. measured the extracellular glutamate level in patients with epilepsy using microdialysis to investigate whether a brain temperature of 15°C shifts the glutamate level. The concentration of glutamate decreased to 66.3% of the precooling levels during the hypothermia period. Also, the reduction in the glutamate level contributes to the reduction in epileptic discharge [52]. Pastukhov et al. discriminated the effects of deep and profound hypothermia on nonpathological and pathological mechanisms of presynaptic glutamate transport by using rat brain nerve terminals. They found that pathological transporter-mediated glutamate release decreased with progressive significance from deep to profound hypothermia [53]. Moreover, one study reported that the stable level of glutamate in the synaptic clefts can sustain synaptic contacts in an active state under hypothermia conditions [54].

## 7. Application of Hypothermia in an Eye Bank

Hypothermia at 2-8°C is the most widely used method for storage of cornea tissue in North America, while in European eye banks organ culture at 28-37°C is usually used because of the extended storage time compared with hypothermia. Corneas stored in an organ for up to four weeks have been shown to retain the integrity of endothelial and epithelial cell layers. Many eye banks prefer not to exceed seven days of storage at 4°C; a limit of four days was typical [2].

### 7.1. Basic and Clinical Study on the Effect of Preservation of Corneal Tissue on the Corneal Endothelium under Hypothermia

Corneal endothelial cells are very important for penetrating keratoplasty (PKP) and endothelial keratoplasty (EK). Price et al. compared endothelial cell loss and graft success six months after endothelial keratoplasty (EK) with paired donor corneas stored in OptiSol GS (commercial whole cornea storage solution) and Life 4°C solutions. The in vivo study showed that endothelial cell loss and graft survival through six months after EK were comparable for a mean of five days in Life 4°C and OptiSol GS. Life 4°C and OptiSol GS are both approved for corneal storage [55]. Mannis et al. investigated the effect of in vivo hypothermic (5°C) perfusion for 30 minutes on corneal endothelial integrity in a cat. The results demonstrated that hypothermic perfusion does not show any effects on the corneal endothelium at the clinical and scanning electron microscope levels [6]. Another study evaluated the influence of temporary hypothermia on porcine corneal endothelial cell density in dextran containing organ-culture medium. Exposure of organ-cultured porcine corneas to 4°C for 12 hours and 21°C for 48 hours does not compromise the endothelial cell density of donor corneas in a clinical relevant manner. Also, lowering the temperature to 4°C for up to 12 hours does not seem to damage the endothelial cell layer [5].

The hypothermic conditions are often employed to maintain the biologics prior to utilization. It is critical to understand the cold-induced changes in tissues. Corwin et al. investigated the role of the unfolded protein response (UPR) in human corneal endothelial cells (HCEC) following hypothermic storage. The unfolded protein response (UPR) is a response to the accumulation of misfolded proteins in the endoplasmic reticulum (ER) of the cell. ER stress and UPR activation responses to hypothermic exposure in the corneal endothelial cells were found. The western blot results showed increases in Bip and endoplasmic oxidoreduction 1-like protein alpha (ERO1-L*α*). The UPR pathway seemed to mediate the caspases, the poly (ADP-ribose) polymerase (PARP), and the mitochondrial-mediated corneal endothelial cell apoptosis. The targeted control of the UPR pathway may improve cell survival and function of HCEC. To this end, UPR appears to be an important pathway. Inhibition of this pathway may improve the HCEC survival for clinical utility [7]. The donor endothelium is the delicate cell layer during storage. Rieck et al. investigated the effect of different concentrations of fibroblast growth factor (FGF-2) added to a modified OptiSol storage medium on endothelial damage after corneal storage at 4°C. The exogenous human recombinant FGF-2 could protect the corneal endothelium from irreversible damage [56].

Corneal endothelial damage restricts the storage time of the cornea under hypothermic conditions. Cultured porcine corneal endothelial cells under 4°C showed morphologic alterations, such as cellular retraction, cellular rounding, and partial detachment. The apoptotic features such as apoptotic bodies, nuclear shrinkage, ruffling of nuclear membranes, and peripheral chromatin condensation appeared during rewarming. Mitochondrial permeability transition is known to be the intrinsic pathway of apoptosis. The losing of mitochondrial membrane potential, long filamentous mitochondria, shortened mitochondria, and small mitochondria are found during exposure to hypothermia and rewarming [57]. One study showed that the mRNA expression levels of core Stk11-p53 signaling factors (Stk11, p53, and p21) in mouse corneal endothelial cells (CECs) were significantly elevated after cryoinjury [58]. The study suggested that cryoinjury led to the damage of mouse CECs by activation of the Stk11-p53 signaling pathway. But the real mechanism of injury is not clear. Moreover, the changes of pH, decreased membrane fluidity, osmotic imbalances, mitochondrial permeability transition pore open, and oxidative stress can also trigger the cell death response.

### 7.2. Preservation of Limbal Stem Cell under Hypothermia

Autologous limbal stem cell (LSC) transplantation can treat a variety of ocular surface diseases, such as recurrent pterygium, chemical injury to the ocular surface, blepharoplasty, and marginal corneal ulcer. Improving the storage of HLECs is important for providing a reliable source of tissue for treating limbal stem cell deficiency. If the limbal tissue taken from the healthy eye is too big, it risks jeopardising the limbal stem cells in the healthy eye [59]. So cryopreservation (4°C) has the potential to indefinitely extend the lifespan of LSCs. However, Raeder et al. found that there was a trend toward a higher apoptotic index of the human limbal epithelial cells (HLECs) with decreased storage temperature. Organ culture storage of cultured human limbal epithelial cells (HLECs) at ambient temperature is superior to organ culture storage at 31°C and OptiSol GS storage at 5°C [60]. Storage at 23°C and 5°C showed the upregulated expression of the antiapoptotic gene BCL2.

### 7.3. The Effect of Preservation of Corneal Tissue on the Corneal Endothelium under Hypothermia

The viability of corneal cells is important for transplantation. By what mechanisms the death of corneal cells occurs is not so clear. Komuro et al. found that both apoptosis and necrosis occurred in cells during corneal storage at 4°C, and apoptosis appeared to account for most cell death in the cornea during preservation. Keratocyte apoptosis was not limited to the anterior stroma, but was more common in the midstroma than anteriorly [61]. This suggested that keratocyte apoptosis during corneal storage at 4°C might be mediated by other mechanisms. If we find a way to inhibit the apoptosis of corneas stored at 4°C, it may prolong the acceptable storage times. The corneal epithelial cells were similar to those of control samples after storage in OptiSol at 4°C for seven days. Extensive epithelial shedding and wing-cell death were observed at 25 days, but nearly 50% of healthy cells were at the basal layer [62]. The effect of freezing and thawing in the biomechanical properties of ex vivo porcine ocular tissue was evaluated. Corneal tissue subjected to freezing at -20°C exhibited significant increases in tangent modulus (mechanical stiffness). However, the increases were insignificant at -80°C. The increases in sclera stiffness at both -20°C and -80°C were insignificant [63]. The underlying causes are not demonstrated, and it may be related to the changes of tissue microstructure.

### 7.4. Preservation of Corneal Tissue and Corneal Endothelial Transplantation

Laaser et al. found that the rebubbling rate of endothelium-Descemet membrane (EDM) grafts was significantly lower in patients receiving organ-cultured (Dulbecco modified Eagle medium) corneas at 34°C as compared with those who received short-term culture (OptiSol GS) at 4°C after Descemet membrane endothelial keratoplasty (DMEK) [64]. Sæthre et al. also found that compared with cold storage solution (OptiSol GS (Bausch & Lomb Inc., Irvine, CA) at 4°C) stored corneas, organ culture solution (containing Minimum Essential M Medium (Invitrogen, Carlsbad, CA) supplemented with Hepes, amphotericin B, and fetal calf serum at 32°C) stored corneas showed lower risk of graft dislocation during Descemet stripping endothelial keratoplasty (DSAEK). The result showed that the type of storage solution might have a decisive role in graft dislocation in DSAEK surgery [65]. They suggested that investigating the biomechanical and immunological properties of the different storage solutions might explain the possible differences in graft adhesion. Due to the temperature difference between cold storage solution (4°C) and organ culture solution (32°C), this may cause differences in corneal endothelial metabolism (such as ATP synthesis and consumption) and corneal endothelial pump function. I suggest that it is necessary to investigate the influence of culture medium on corneal biomechanics, corneal stiffness, corneal collagen changes, and corneal immunogenicity.

### 7.5. Preservation of Corneal Tissue and Infection

The incidence of postkeratoplasty graft-transmitted fungal infections was reported in the eye bank. Tran et al. reported that donor corneas exposed to amphotericin B ≤ 2.59 *μ*g/mL were safe for the cornea endothelium. The corneal endothelium cell mitochondrial function and viability did not differ in donor corneas compared with the controls [66]. This result showed the efficacy and safety of suitable amphotericin B concentrations on *Candida albicans* in cold storage conditions. Reitberger et al. reported that argon cold plasma treatment could reduce or eliminate a wide range of common pathogens responsible for corneal infections without impairing corneal epithelial cells [67].

Hypothermia not only protects and preserves corneal tissue but also brings local cold damage. Corneal endothelial damage occurred during hypothermic storage. This study investigated the cultured porcine corneal endothelial cells exposed to 4°C in either the cell culture medium, Krebs-Henseleit buffer, OptiSol GS solution, or McCarey-Kaufman medium for five hours to 14 days and then rewarmed under cell culture conditions (three hours). The corneal endothelial cells showed 47% ± 8% and 64% ± 20% cell death following cold storage and rewarming in cell culture medium [57]. So the safety, quality, and biological activity of the corneal endothelial cells under hypothermic conditions still need further study.

## 8. Application of Hypothermia in Corneal Refractive Surgery

The cooling of the corneal surface before, during, and after excimer laser photorefractive keratectomy is a safe and effective method to reduce postoperative pain, subepithelial haze, and myopic regression. Cooling the cornea after laser ablation might lessen the release of some chemical mediators such as prostaglandins and substance P and also decrease the inflammatory reaction [68]. Another study also reported that haze was significantly reduced in myopia eyes between -6.00D and -9.75D by cooling the cornea using a chilled balanced solution intraoperatively and immediately after ablation during photorefractive keratectomy [69]. But so far, the mechanism by which low temperature reduces the formation of haze is unclear.

## 9. Application of Hypothermia in Allergic Conjunctivitis

The application of cold compress on the ocular surface can cause vasoconstriction and then reduce hyperemia and relieve signs and symptoms in acute seasonal conjunctivitis. Bilkhu et al. found that artificial tears combined with cold compress reduced hyperemia more than other treatments [70]. Cold compress can enhance the effectiveness of pharmacological therapy on the signs and symptoms of allergic conjunctivitis [71]. Cold compresses can reduce the release of inflammatory substances, thereby reducing symptoms, but it is important to remove allergens to treat allergic conjunctivitis.

## 10. Hypothermia and Vitrectomy

The temperature changes of the vitreous and retinal surface during surgery were described. Before vitrectomy, the mean vitreous temperature was 33.9°C and retinal temperature was 34.8-35.2°C. During vitrectomy, the mean vitreous temperature was 24.9°C and retinal temperature was 28.4-29.5°C [72]. These results suggested that the vitreous and retina are cooled to much lower temperatures during vitreous surgery.

Local hypothermia during vitreous surgery under fluctuating intraocular pressure could inhibit the breakdown of the blood-aqueous barrier [73]. Hypothermia may be a promising strategy for reducing postoperative inflammation in the early stages. Rinkoff et al. investigated whether reducing the intraocular temperature by cooling the infusion fluid during vitreous surgery could protect the retina and retinal pigment epithelium from light damage. The results showed that light exposure during the infusion of room temperature (22°C) prevented the clinical and histologic damage seen in the body temperature fluid (39°C). Also, compared with light exposure during infusion of body temperature fluid (39°C), room temperature (4°C) prolonged the duration of damage-free light exposure time [74]. Chen et al. examined the mechanical characteristics of the retina at a low temperature. The transition modulus and the transition stress are significantly different between 37.0°C and 26.1°C and between 37.0°C and 7.8°C [75]. Lowering the temperature of the retina can increase the resistance of the retina, and it is useful to decrease retinal damage in posterior eye surgeries. However, research has not yet come to a conclusion as to what extent the temperature can be lowered without damaging the retina. One study suggested that mild intraocular hypothermia (22°C) perfusion with lactated Ringer's solution was safe in rabbit eyes which had undergone vitrectomy. They suggested that the use of moderately cooled infusion fluids during vitreous surgery might be appropriate and that the extreme cooling (4°C) was not advisable [76].

Indocyanine green (ICG) is usually used to stain the inner limiting membrane (ILM) during macular surgery. Kunikata et al. investigated the toxic effect of ICG on the cultured human retinal pigment epithelial (ARPE-19) cells and whether hypothermia could protect the ARPE-19 cells against the ICG toxicity. They found that high concentrations and long exposure times of ICG were toxic and hypothermia of 4°C could protect the toxicity of ICG [77]. Trypan blue (TB) has also been used to stain the transparent epiretinal membrane (ERM) during macular surgery. Another study showed that TB was toxic to cultured ARPE-19 cells, and the toxicity was dose and exposure time dependent. Exposing the cells at 4°C had a protective effect against TB toxicity [78].

## 11. Hypothermia and Retinal Ischemia

### 11.1. The Protective Effect of Hypothermia on Ischemic Retina

Ischemia for periods of 90 min or less produced mild retinal tissue damage while 120 min or more produced severe damage [79]. Hypothermia protects the retina from tissue damage in temporary retinal ischemia in rats. Short ischemic durations of less than one hour at body temperature (37°C) are less harmful to the ganglion cells; after four hours of ischemia, there is no detectable activity. However, under hypothermia conditions, ganglion cell activities could still be detected at the longest time durations of 12 hours at 21°C and 50 hours at 4°C. Hypothermia might be a method for the treatment of retinal ischemia [80]. The hypothermia on the retinal tolerance of ischemia has been confirmed. A low temperature does indeed lead to a pronounced increase in retinal ganglion cell survival time.

Global or ocular hypothermic preconditioning (HPC) (33°C and 32°Cfor 20 min, respectively) applied 24 h before ischemia significantly preserved retinal function (electroretinography) in eyes exposed to ischemia/reperfusion injury. In animals submitted to global HPC, a significant preservation of the ERG a-wave, b-wave, and oscillatory potentials (Ops) amplitude was observed, whereas ocular HPC significantly prevented the decrease in the amplitude of ERG b-wave and Ops [81]. Ocular HPC significantly prevented the decrease of ERG b-wave and Ops amplitude and prevented glutamate-induced alteration in the retinal structure. Tamai K et al. used hypothermic irrigation solutions (8°C, 22°C, and 38°C) during vitrectomy in pressure-induced ischemic rabbit eyes. They examined the parameters of electroretinograms, glutamate levels in the vitreous, and grading of retinal damage. They found that the recovery rate of a-wave amplitude in the eyes perfused at 38°C was significantly lower than at 8°C and that of b-wave amplitude was significantly lower in the eyes perfused at 38°C than in either 8°C or 38°C [82].

### 11.2. The Protective Mechanism of Hypothermia on Ischemic Retina

The mechanisms through which hypothermia protects the ischemic retina are not clear. But there are some assumptions, namely, decrease in metabolism, the breakdown of the blood-aqueous barrier, and hypothermia-reduced neurotransmitter release. Hypothermia during retinal ischemia was effective in protecting retinal neuronal death. Wang et al. found that lowering the temperature to 29°C could reduce DNA fragmentation in the retina after transient retinal ischemia [83].

Retinas treated with mild or moderate hypothermia during ischemia showed attenuation of glucose utilization and decrease in the rate of lactate production compared with normothermic retinas. On the other hand, during return-to-control conditions, there was a significantly greater recovery of glucose utilization and lactate production in the retinas treated with mild or moderate hypothermia than in the normal retinas. The increased metabolic activity may reflect the return of normal cellular function after hypothermia. Quiñones-Hinojosa et al. demonstrated that hypothermia protected against ischemic injury in the retina. The mechanism by which hypothermia protects the retina may be attributed to its having a larger effect on energy requirements than energy production. As the electrophysiological activity of retinal nerve cells decreases under low temperature and the demand for energy decreases, it has been shown that hypothermia of 31°C inhibited the depletion of adenosine triphosphate (ATP) during hypoxia-ischemia in newborn rats [84].

The distribution of TUNEL-positive cells is not equal among different retinal areas and layers in the rat retina following transient ischemic injury induced by thrombotic occlusion-thrombolytic reperfusion. The parapapillary and central areas of the retina were affected to the highest degree, but the outer nuclear layer was found to be almost intact [85]. The reason may be due not only to the blood supply but also that the outer nuclear layer (ONL) neuronal cells are less sensitive to ischemic damage.

Tamai et al. found that the vitreous glutamate level at 30 minutes was significantly higher than in both the 8°C and the 22°C groups. So, they suggested that the significant increase of glutamate levels in the 38°C group was thought to be primarily due to ischemic injury [82]. Salido et al. also reported that the intravitreal injection of supraphysiological levels of glutamate could induce alterations in retinal function and histology [81].

## 12. Hypothermia and Retinal Hypoxia

The retinal ganglion cells (RGCs) are the first cells from the retina that suffer from hypoxia. A mild reduction of temperature could protect the RGCs against damage [86]. Hypothermia could function as a promising therapeutic option for hypoxic diseases.

The thickness of the inner retina including the ganglion cell layer, the optic nervous fiber layer, and the inner limiting layer was increased after perinatal asphyxia (PA). Hypothermia (15°C) during perinatal asphyxia (PA) prevented cellular and morphological alterations and transformed such changes in the retina [87]. Rey-Funes et al. reported that hypothermia prevented astrogliosis reaction and development of neovascularization in the inner retina. If hypothermia could be translated into the clinics, it could be an efficient tool to prevent the development of retinal neural damage and angiogenesis due to global hypoxia-ischemia [87].

Nitric oxide (NO) is increased in the retina after hypoxia-ischemia [88]. NO causes retinal damage by altering the catalytic activity of many enzymes resulting in a neurotoxic effect [89]. Cells of the most inner layers of the retina are sensitive to oxygen deprivation. Both inducible and constitutive NO synthase activity is significantly increased in perinatal asphyxia (PA). The hypoxia-ischemia might induce alterations in the nitric oxide system [90].

## 13. Hypothermic Protection of Retinal Nerves

Hypothermia has a strong neuroprotective effect on the hypothermic retinal explants. Hypoxia-induced retinal thinning and the decreased survival of retinal ganglion cells (RGCs) were vigorously attenuated by hypothermia of 20°C [86]. Under ischemic conditions, the inner retinal neurons are more vulnerable than the outer retinal photoreceptors. Ischemia of 60 min caused not only neurodegeneration in the inner retinal layer but also damage to photoreceptor cells in the outer nuclear layer. Ischemia of 45 min mainly caused neurodegeneration in the inner retinal layer. Hypothermia (33°C) reduced the cell loss in the ganglion cell layer under retinal ischemia induced by intraocular pressure to 130 mmHg for 45 min [9].

### 13.1. The Protective Mechanism of Hypothermia on the Retinal Nerve

The exact mechanism of hypothermia-induced neuroprotection is still unclear. Sun et al. demonstrated that hypothermia (32°C) showed significant neuroprotection in photoreceptors against glucose deprivation- (GD-) induced injury and visible light-induced retinal damage of mice in vivo. Also, the study suggested that hypothermia promoted neuroprotection in photoreceptors via activation of the cold-inducible RNA-binding protein (Cirbp) pathway and inhibited the activation of poly (ADP-ribose) polymerase-1 (PARP-1) in light-damaged retinas [8].

Cold-shock proteins (CSPs) were localized in the cytoplasm of ganglion cells, Müller cells, horizontal cells, cone bipolar cells, photoreceptors, and the RPE. The extensive localization implies that CSPs may exert a profound impact on the physiology of the mammalian retina when exposed to hypothermia. Larrayoz et al. found that expression of CSP including RNA-binding motif protein 3 (RBM3) and cold inducible RNA-binding protein (CIRP) was upregulated in the mammalian retina following exposure to hypothermia in a cell type-specific pattern [91].

Hydrogen peroxide- (H_2_O_2_-) induced oxidative stress of the ex vivo model of cultivated porcine retinas can be counteracted or alleviated through mild hypothermia (30°C) treatment. Mueller-Buehl et al. found that hypothermia reduced proceeding apoptosis and Bax/Bcl-2 ratio in the retina leading to increased ganglion cell survival [92]. Hypothermia reduced the Bax/Bcl-2 ratio, which is only involved in intrinsic apoptosis. So, they suggested that hypothermia particularly inhibited the intrinsic pathway. Also, hypothermia treatment rescued the amacrine cells and reduced the microglia and microglia reaction.

Aberrant angiogenesis and exacerbated gliosis are factors related to the increased thickness of the inner retina as a result of hypoxia-ischemia. Hypothermia prevents the aberrant angiogenesis and exacerbated gliosis in the perinatal asphyxia (PA). The neuroprotective effect of hypothermia may be the inhibition of nitric oxide (NO) synthase activity and protein nitration [93].

## 14. Application of Hypothermic Technology in Other Fields of Ophthalmology

Kataoka et al. investigated whether cooling the anterior ocular segment by using contact lenses with a cooling system during laser iridotomy prevented corneal damage in rabbits. The results showed that cooling inhibited corneal damage after laser iridotomy [94]. However, the mechanism of the protection by cooling is not clear.

The cooling method may be a useful approach for relieving surgical trauma. Fujishima et al. tried to investigate the effect of cooling on the eyes after cataract surgery by using a special ice-cold eye mask. Cooling reduced postoperative pain and inflammation after cataract surgery, with no adverse effects [95].

## 15. Application of Cryotherapy for the Retina during Vitreoretinal Surgery

Retinal cryotherapy is used to eliminate ischemic retina and retinal vessels and avoid excessive VEGF production due to hypoxia before. Retinal cryotherapy is often used in retinal hole, retinal tears, and diabetic retinopathy during surgery. But now the intravitreal injection of anti-VEGF drugs is more widely used.

Anti-VEGF combined with retinal cryotherapy or laser coagulation can effectively treat stage 3 Coats' disease [96]. However, cryotherapy can increase the proliferation of the retina and the occurrence of traction. Also, cryotherapy for retinopathy of prematurity can cause retinal folds, reduced foveal depth, and absence of a foveal pit with poor visual acuity [97].

In the diabetic eye, retinal ischemia is one of the important factors contributing to the formation of retinal neovascularization. Panretinal photocoagulation is hard to access the most peripheral retina, which is the remaining retinal ischemic area. Anterior peripheral retinal cryotherapy combined with cryotherapy of sclerotomy sites during vitrectomy might be adjunct procedures to inhibit fibrovascular ingrowth and prevent recurrent vitreous hemorrhage [98]. In the treatment of proliferate diabetic retinopathy (PDR) with vitreous hemorrhage, intravitreal bevacizumab injection combined with anterior retinal cryotherapy can get rapid vitreous clearance and improved visual acuity compared with bevacizumab monotherapy [99]. However, Entezari et al. reported that cryotherapy of the sclerotomy sites did not reduce the risk of late postvitrectomy hemorrhage in diabetic eyes [100]. The reason might be an increased release of inflammatory and angiogenic mediators caused by cryotherapy-induced blood-retinal barrier breakdown.

## 16. Conclusions and Future Directions

Hypothermic technology has been widely used in medicine. The protective mechanism of hypothermia on tissue is still unclear. The application of hypothermic technology in ophthalmology is mainly concentrated on the eye bank, intraocular surgery, and some ocular surface diseases. However, the practical applications of hypothermic technology in ophthalmology are very few.

The protective effect of hypothermia on the eyes needs further study. Aspects could include the effect of hypothermia on corneal nerves, the protective mechanism of hypothermia on the retina, the effect of hypothermia on ganglion cell transmission function, and the effect of hypothermia on the function of retinal visual rod cells and visual cone cells. The retina is rich in nerves and blood vessels, so some systemic diseases can cause retinal ischemia and hypoxia. Hypothermia can temporarily reduce the oxygen demand of the retina. It would be of great significance to study the protective mechanism of low temperature on the retina. It may also be used for retina transplantation in the future.

Therapeutic hypothermia has been shown to be neuroprotectant in labs studying different disease models or organ protection. However, there are still challenges in applying this method routinely to each pathological condition. There are still clinical trials to be overcome, such as the upregulation of several cytoprotective genes under hypothermia, the suitable temperature for different organ storage, and the harmful side effects of low temperature. Gene expression analysis during ischemia or gene expression changes occurring during hypothermic treatment are important for studying the related proteins which play a role in tissue damage and which are important for hypothermic neuroprotection.

## Data Availability

The data used to support the study can be available upon request to the corresponding author.
